# The Impact of the Angulus Biopsy on the Detection of Staging and the Grading of Chronic Gastritis

**DOI:** 10.3390/diagnostics13182928

**Published:** 2023-09-13

**Authors:** Sergey G. Khomeriki, Dmitry S. Bordin, Natalia M. Khomeriki, Elena V. Parfenchikova, Karine A. Nikolskaya, Valeria A. Ivanova, Margarita V. Chebotareva, Maria L. Gretskaya, Irina N. Voynovan, Mariia A. Kiriukova, Maria A. Livzan, Igor E. Khatkov

**Affiliations:** 1 A.S. Loginov Moscow Clinical Scientific Center, 111123 Moscow, Russia; xomep@mail.ru (S.G.K.); e.bystrovskaya@mknc.ru (E.V.P.); gastro2@yandex.ru (K.A.N.); chebotareva.rita.92@mail.ru (M.V.C.); firj@mail.ru (M.L.G.); irinavmgd@mail.ru (I.N.V.); ihatkov@gmail.com (I.E.K.); 2 Department of Outpatient Therapy and Family Medicine, Tver State Medical University, 170100 Tver, Russia; 3 Department of Propaedeutic of Internal Diseases and Gastroenterology, A.I. Yevdokimov Moscow State University of Medicine and Dentistry, 127473 Moscow, Russia; 4 M.F. Vladimirsky Moscow Regional Clinical Research Institute (MONIKI), 129110 Moscow, Russia; nataliakhomeriki@gmail.com; 5 Research Institute for Healthcare Organization and Medical Management of Moscow Healthcare Department, 115088 Moscow, Russia; 6 Department of Internal Medicine and Gastroenterology, Omsk State Medical University, 644099 Omsk, Russia; mlivzan@yandex.ru

**Keywords:** chronic gastritis, gastric atrophy, OLGA system, gastritis staging, incisura angularis

## Abstract

There is a generally recognized need for a morphological assessment of the individual risk of developing gastric cancer in a patient with chronic gastritis, according to the OLGA system (Operative Link for Gastritis Assessment). At the same time, the role of assessing the biopsy from the incisura angularis remains controversial. The aim of our study was to assess the value of incisura angularis biopsy in staging gastritis according to the OLGA system by examining the atrophic and inflammatory changes in the antrum, incisura angularis, and body. Materials and Methods: A total of 718 patients (576 women and 142 men) aged 20 to 84 years were examined. Most of the patients were in the age group of 50 to 70 years (54.6%). Depending on the detection of *H. pylori* and autoimmune gastritis markers, all patients were divided into three groups. The first group included 380 patients with *H. pylori* gastritis without signs of autoimmune gastritis. The second group consisted of 209 patients with autoimmune gastritis, in whom no infection was detected during the examination, and there were no indications of *H. pylori* eradication. The third group consisted of 129 patients with chronic gastritis of combined etiology (autoimmune and *H. pylori*). Endoscopy biopsies were taken according to the updated Sydney System. Histological assessments of the grade and the stage of gastritis were carried out according to the standard OLGA-based protocol. Then, the same assessments were evaluated without taking into account histological changes in the incisura angularis. Results: When assessing the severity of inflammatory changes in the gastric mucosa according to the OLGA system, grade II (72.3%) was most often detected in all groups of patients. A severe degree of activity of chronic gastritis was most often observed in the group of patients with *H. pylori* gastritis (6.1%). These indicators practically did not change if the assessment did not take the angulus biopsy into account. When assessing the severity of atrophy of the glands in the gastric mucosa in patients of the first group, mild stages of atrophy prevailed. Without taking into account the angulus biopsy, a decrease in the stage of atrophy was observed in 27 cases (7.11%), and in only 4 cases did stage IV change to stage III, while in 23 cases, discrepancies were noted only within groups with a mild stage of atrophy. There were no transitions from stage III to stage II. In the group of patients with autoimmune gastritis, pronounced stages of atrophy prevailed—in more than 77%. Without taking into account the angulus biopsy, a decrease in the stage of atrophy was observed in eight cases (3.83%), and in three (1.4%) patients, stage III was changed to stage II. In the group of patients with combined etiology (autoimmune + *H. pylori*), severe stages of atrophy also prevailed (70.5%). A decrease in the stage of atrophy without taking into account the angulus biopsy was only observed in three patients (2.32%), of which two cases concerned patients with mild stages of atrophy. Thus, in general, severe stages of atrophy of the gastric mucosa (stages III and IV according to the OLGA staging system) were detected in 313 patients (43.59%). If the assessment of the atrophy stage did not take into account changes in the angulus biopsy, then severe stages of atrophy (III and IV according to OLGA) were detected in 310 patients (43.17%). In total, changes in the assessment of the atrophy stage occurred in 38 patients (5.29%), and this was more often observed in patients with stages I and II of atrophy. Conclusions: Accounting for histological changes in the incisura angularis does not significantly affect the assessment of the grade and stage of chronic gastritis according to the OLGA system, regardless of the etiology of atrophic gastritis.

## 1. Introduction

Chronic atrophic gastritis is considered a preneoplastic disease, the stage of which determines the individual risk of developing gastric cancer and the need for dynamic endoscopic monitoring [1]. The most common cause of chronic gastritis is *H. pylori* infection, which causes more than 90% of gastric cancers worldwide [2,3]. To assess the grade and stage of gastritis, the OLGA system is used [3], which provides a morphological assessment of five biopsies of the gastric mucosa, taken according to the updated Sydney System (two biopsies from the antrum, two biopsies from the corpus, and one biopsy from the incisura angularis) [4,5].

The clinical value of additional biopsies from the incisura angularis remains unclear and controversial. According to one data source, a routine biopsy of the incisura angularis would provide little additional clinical information to that obtainable from antrum and body biopsies [6,7]; for others, the incisura angularis undergoes more severe atrophic, metaplastic, and chronic inflammatory changes than the antrum and body [5]. That is why incisura angularis biopsies should be routinely included in the biopsy sampling protocol [8].

In line with MAPS II recommendations, biopsies should be taken from at least two topographic sites (antrum and body). At the same time, if the OLGA or OLGIM systems are used to stratify patients with atrophic gastritis, an additional biopsy of the incisura should be considered. For patients with mild to moderate atrophy restricted to the antrum, there is no evidence to recommend surveillance [9].

The high incidence of gastric cancer in Russia and the poor prognosis, which usually reflects the late stage of diagnosis of this potentially preventable and curable cancer, justifies the need to develop a strategy for primary and secondary prevention. To this end, from 2022 to 2024, a study of the prevalence of *H. pylori* and precancerous changes in residents of the city of Moscow is being conducted.

The purpose of this article is to analyze the impact of an additional biopsy from the incisura angularis on OLGA gastritis staging.

## 2. Materials and Methods

### 2.1. Patient Data

We conducted an observational epidemiological prospective study. The protocol of the study was registered at ClinicalTrials.gov (NCT05775120).

In total, 718 patients were examined: 576 women (80%) and 142 men (20%) aged 20 to 84 years (mean age 54.6 +/− 12.8 years). Most of the patients were aged 50 to 70 years (54.6%). The study included patients who were examined at the A.S. Loginov Moscow Clinical Scientific Center from 2017 to 2023, including respondents from the *H. pylori* Prevalence and Precancerous Changes Study in Moscow Residents (2022–2023).

All of the patients were tested for *H. pylori* using at least one of the following methods: histological method, rapid urease test, serological test *(H. pylori* IgG), determination of monoclonal antigen in feces, ^13^C urease breath test [10,11], as well as data on the presence of infection in medical records. All respondents of the study on the prevalence of *H. pylori* and precancerous changes in Moscow residents underwent a ^13^C urease breath test and an assessment of antibodies to *H. pylori* IgG (Gastropanel, Biohit). Gastritis was considered to be associated with *H. pylori* if infection was detected using one of the above methods; it was detected in 509 patients (70.9%).

Autoimmune gastritis was detected in the presence of antibodies to parietal cells or intrinsic factors of Castle in the blood, as well as on the basis of histopathological changes in the gastric mucosa of the body (e.g., emperipolesis or the destruction of parietal cells) [12].

Autoimmune gastritis was detected in 338 patients (47.1%), of which 129 patients (18%) had *H. pylori* and histological signs of *H. pylori* gastritis.

All examined patients underwent endoscopy with the sampling of 5 biopsies of the gastric mucosa according to the updated Sydney System. A histological assessment of the degree of inflammation activity and the stage of atrophy was performed according to the standard OLGA protocol. Changes in the antral and body mucosa, as well as in the area of incisura angularis, were evaluated. Then, the same parameters were evaluated without taking into account histological changes in the incisura angularis.

**Figure 1:**
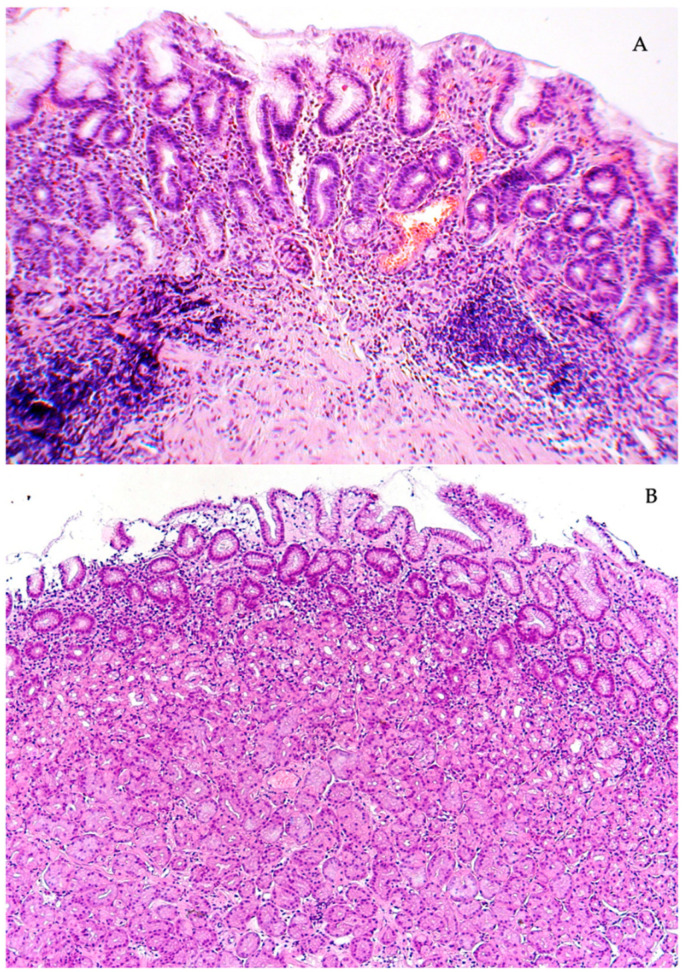
Chronic *H. pylori* gastritis. (**A**) Active inflammation of the antrum. Atrophy of the terminal sections of the pyloric glands. (**B**) Superficial gastritis of the body of the stomach. End sections of the main glands of the usual structure. Stained with hematoxylin and eosin. Magnification: (**A**) ×120, (**B**) ×120.

**Figure 2:**
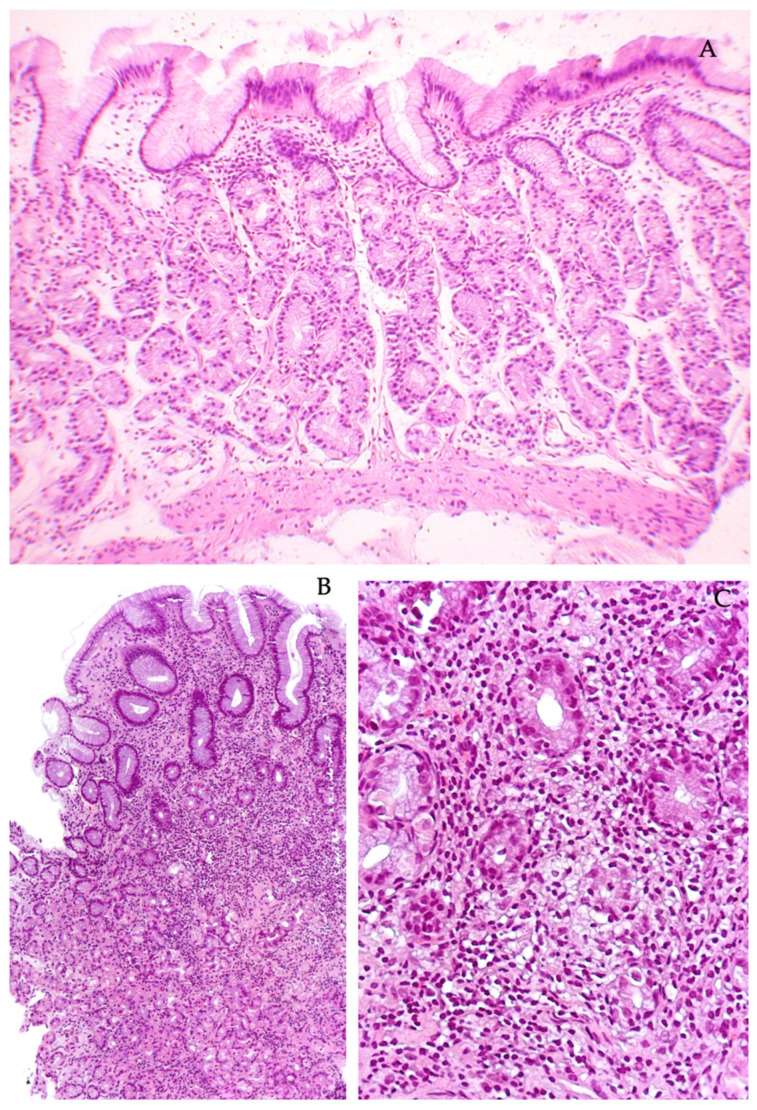
Chronic autoimmune gastritis. (**A**) Histological structure of the pyloric glands of the antrum of the stomach without changes. (**B**) Areas of periglandular lymphocytic infiltration in the mucosa of the body of the stomach. (**C**) Destruction of the terminal sections of the main glands. Stained with hematoxylin and eosin. Magnification: (**A**) ×120, (**B**) ×120, (**C**) ×300.

**Figure 3:**
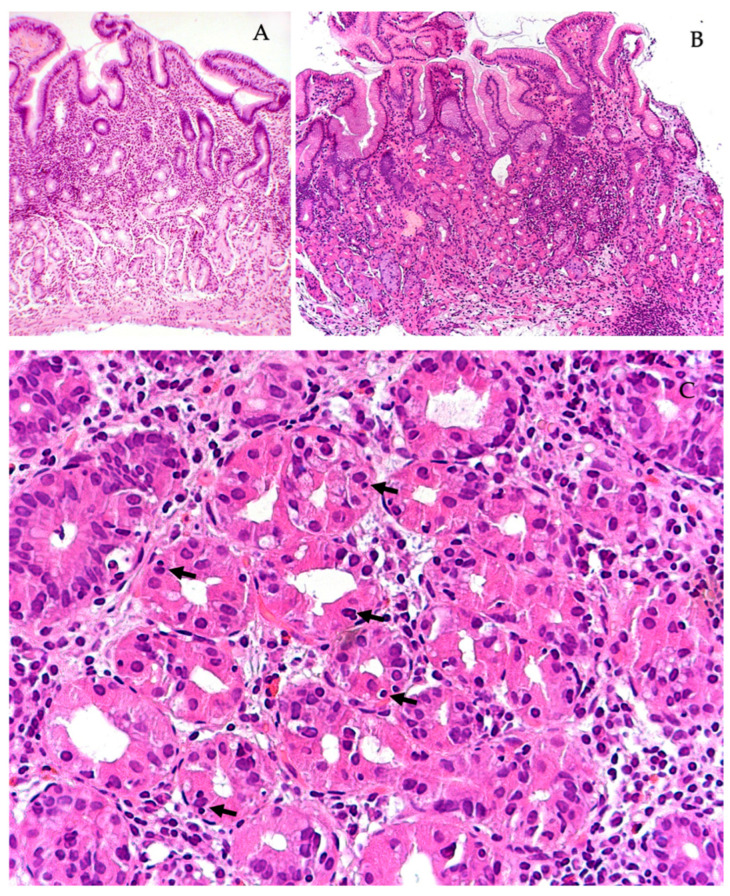
Chronic *H. pylori* gastritis in combination with autoimmune gastritis. (**A**) Chronic inflammation of the antrum with focal atrophy of the pyloric glands. (**B**) Focal atrophy of the terminal sections of the main glands in the mucous membrane of the body of the stomach. (**C**) Emperipolesis (arrows) of individual parietal cells in the mucosa of the body of the stomach. Stained with hematoxylin and eosin. Magnification: (**A**) ×120, (**B**) ×120, (**C**) ×500.

Based on the results of examination for *H. pylori* and markers of autoimmune gastritis, all patients were divided into 3 groups (Table 1). The first group included 380 patients with chronic gastritis associated with *H. pylori* without signs of autoimmune gastritis (Figure 1). The second group consisted of 209 patients with autoimmune gastritis, in whom the examination was not detected, and there were no indications for the treatment of *H. pylori* in the anamnesis (Figure 2). The third group consisted of 129 patients with chronic gastritis of combined etiology—with markers of the presence of *H. pylori* infection and autoimmune gastritis (Figure 3). *H. pylori* in groups 1 and 3 was detected using the methods presented in Table 2.

### 2.2. Statistical Analysis

Statistical processing was carried out using the SPSS program (version 13.0). Statistical analysis included checking the nature of the distribution of indicators. The data obtained in the tables and text are presented as absolute and relative values (*n*, %).

To assess the significance of differences in diagnostic approaches, various statistical methods were used: count of the Kappa coefficient of agreement (according to Cohen), sensitivity and specificity, and Chi-square test.

## 3. Results

When assessing the severity of inflammatory changes in the gastric mucosa according to the OLGA system, grade II (72.3%) was most often detected in all groups of patients (Table 3).

A severe degree (OLGA IV) of inflammation was most often observed in the group of patients with *H. pylori*-associated chronic gastritis (6.1%). These indicators practically did not change if the assessment did not take into account the biopsy from the incisura angularis.

When assessing the severity of atrophy of the glands in the gastric mucosa in patients of the first group, mild stages of atrophy prevailed (Table 4). In more than a third of cases, atrophy of the gastric glands was not detected. Without taking into account the biopsy from the incisura angularis, a decrease in the stage of atrophy was observed in 27 cases (7.11%), and in only 4 cases did stage IV change to stage III, while in 23 cases, discrepancies were noted only within groups with a mild stage of atrophy. There were no changes from stage III to stage II.

In the group of patients with autoimmune gastritis, severe stages of atrophy (OLGA III–IV) prevailed in more than 77% (Table 5). Without taking into account the biopsy from the incisura angularis, a decrease in the stage of atrophy was observed in eight cases (3.83%), and in three (1.4%) patients, stage III was changed to stage II.

In the group of patients with combined etiology (autoimmune gastritis in combination with *H. pylori* chronic gastritis), severe stages of atrophy (OLGA III–IV) also prevailed in more than 70.5% (Table 6).

A decrease in the stage of atrophy without taking into account a biopsy from the incisura angularis was only observed in three patients (2.32%), of which two cases concerned patients with mild stages of atrophy.

Thus, in general, severe stages of atrophy of the gastric mucosa (OLGA III and IV) were detected in 313 patients (43.59%). If the assessment of the stage of atrophy did not take into account changes in the incisura angularis, then severe atrophy stages (OLGA III and IV) were detected in 310 patients (43.17%). In total, changes in the assessment of the atrophy stage occurred in 38 patients (5.29%), and this was more often observed in patients with stages I and II of atrophy.

The Kappa coefficient of agreement (Cohen) was calculated for two diagnostic approaches for detecting severe stages of atrophy (OLGA III and IV): with and without taking into account the biopsy from the incisura angularis. Kappa coefficient = 0.991, SE of Kappa = 0.005; 95% confidence interval: from 0.982 to 1.000. Kappa between 0.81 and 1.00: almost perfect agreement (very high level of agreement between the results of the two diagnostic approaches).

If the results of detection of severe stages of atrophy (OLGA III and IV) taking into account the biopsy from the incisura angularis are taken as the “gold standard” and the operational characteristics of the diagnostic test are calculated without taking into account the biopsy from the incisura angularis, then all indicators will be comparable with the results obtained taking into account incisura angularis biopsies: sensitivity 99.04% (95% CI 97.22% to 99.80%), specificity 100.00% (99.09% to 100.00%), and accuracy 99.58% (98.78% to 99.91%).

If we compare the two approaches using the Chi-square test, then for a four-field table, the following result will be obtained: Chi-squared: 0.0255 (DF 1, Significance level *p* = 0.8731). This also indicates the absence of statistically significant differences in these diagnostic approaches.

## 4. Discussion

Russia is one of the countries with a high incidence of gastric cancer [13] and a high prevalence of *H. pylori* [14], which makes it important to develop strategies for the primary and secondary prevention of gastric cancer.

According to the European MAPS II guidelines, it is recommended to take two biopsy tissue samples from the body and the antrum of the stomach. In this case, the sampling of material from the incisura angularis is not mandatory [9]. In support of the need to take material from this additional point, studies show the frequent localization of intestinal metaplasia foci in the area of the incisura angularis [15,16].

In the updated Sydney System, the protocol involves taking biopsy material from at least five points: two from the antrum (along the lesser and greater curvature, 3 cm from the pylorus); one from the incisura; and two from the body of the stomach (along the lesser curvature, 4 cm proximal to the angle of the stomach and in the middle of the line along the greater curvature). This approach differs from the original Sydney one, with the collection of two fragments from the body and two from the antrum [4]. According to a large study analyzing the material of 400,738 biopsy studies, the effectiveness of this approach in relation to diagnosing *H. pylori* infection and the detection of intestinal metaplasia was established [17].

Studies are being carried out to evaluate the diagnostic efficiency of taking biopsy material from additional sites/points of the gastric mucosa. In particular, there is a point of view on the minimum value of taking material from the incisura angularis in terms of an adequate verification of morphological changes. Several studies have been published evaluating the role of accounting for the incisura angularis in the OLGA staging of atrophy, with conflicting results. In a study by Eriksson N.K. et al. in Finland on 272 patients, only 11 of them (4.0%) showed signs of chronic inflammation exclusively in the biopsy material from the incisura angularis. Of all the 272 patients studied, changes in the angle of the stomach were detected in 120 cases. At the same time, isolated registration of intestinal metaplasia, which contributes to the assessment of chronic gastritis stage, was only registered in the material from the incisura angularis in 13 patients (4.7%). Based on the data, the authors concluded that biopsy material from the incisura angularis is of low diagnostic value, provided that the study is carried out according to the standard Sydney protocol with sampling from two parts of the body and the antrum of the stomach. [6].

In a German study of 213 patients, it was found that, in the absence of biopsy material from the incisura angularis, 8% of cases of atrophic gastritis and 3% of cases of gastritis with intestinal metaplasia would be undiagnosed. Despite the unconditional contribution of incisura angularis material to the total assessment of the chronic gastritis stage, especially with mild atrophy, the impact on the identification of pronounced changes (stages ΙΙΙ–ΙV) was minimal [18].

In Stolte M. et al.’s study on the material of 328 patients, no significant contribution of biopsy material from the incisura angularis to the assessment of atrophy, intestinal metaplasia, and the degree of lymphocytic infiltration of the gastric mucosa was demonstrated. However, the histological characterization of the gastric angle zone may be of interest in itself. In particular, it was found that the presence of the antral type of mucosa in the area of the incisura (all glands of the antral type) was associated with more pronounced signs of chronic gastritis, primarily in the antrum [19]. Thus, the fact that antralization of the incisura angularis can be considered as an additional characteristic of the evolution of chronic gastritis serves as a kind of additional marker for assessing the duration and severity of the inflammatory process, therefore acting as a surrogate (indirect) indicator of the stage of chronic gastritis. In a study by Rubio C.A. et al., the possibility of just such a diagnostic interpretation of antralization of the stomach angle using the immunohistochemical detection of cells expressing gastrin was shown in principle [20]. It is interesting that the occurrence of this phenomenon is primarily associated with inflammation and does not depend on the presence of *H. pylori* colonization and the etiology of gastritis in general, which was also noted in our study.

In Xia H.H. et al.’s study conducted in 2000, an interpretation was proposed of the origin of antralization of the stomach angle, involving the multidirectional migration of hyperplastic mucin-producing cells of the isthmus of the glands in the upper and lower directions with the replacement of more specialized parietal and chief cells of the fundic glands [21]. Currently, the most common point of view about the origin of such antralization involves the direct transdifferentiation of mature chief cells into mucin-producing pseudopyloric cells with the expression of trefoil factor 2 with the advent of a special cell line—spasmolytic polypeptide-expressing metaplasia (SPEM) [22].

Zhang C et al. demonstrated that the material from the incisura angularis is more indicative for the verification of atrophy and intestinal metaplasia than even the classical sampling of material from the body and antrum of the stomach [23]. These data are similar to the results obtained by Isajevs S. et al. in a study of 835 patients, where it was found that severe atrophy/intestinal metaplasia and signs of chronic inflammation were more common in the incisura angularis than in the body and antrum, to the point where assessing the stages of high risk of developing gastric cancer (stages ΙΙΙ–ΙV of chronic gastritis OLGA/OLGIM) excluding the point of the incisura angularis would be underestimated by 30–35% [8].

The data presented by us were obtained in Moscow during the examination of 718 patients. In a morphological study, taking into account the incisura angularis, severe stages of atrophy of the gastric mucosa (stages III and IV according to the OLGA system) were detected in 313 patients (43.59%). If, when assessing the stage of atrophy, changes in the area of the incisura angularis were not taken into account, severe stages of atrophy (III and IV according to OLGA) were detected in 310 patients (43.17%). In general, changes in the assessment of the stage of atrophy were noted in 38 patients (5.29%); however, this was mainly observed in patients with stages I and II, which is consistent with studies by other authors [23].

The stratification of the risk of developing gastric cancer is based primarily on the assessment of morphological changes in the gastric mucosa according to the OLGA system, proposed by an international group of experts in 2008. The stage of gastritis, determined integrally by the prevalence and severity of atrophy, is a marker of the individual risk of developing gastric cancer. From a practical point of view, it is extremely important to identify a cohort of patients with the highest possible risk of developing gastric cancer, that is, with severe atrophy of the gastric mucosa (OLGA III–IV) [24], and subsequently, when analyzing the minimum sufficient number of gastrobiopsy specimens to accurately determine the stage of gastritis.

The leading etiological factors of chronic atrophic gastritis are *H. pylori* infection and autoimmune inflammation of the gastric mucosa (autoimmune gastritis). In our study, there was no task to assess the epidemiology of autoimmune gastritis, and the higher incidence of autoimmune gastritis in the research cohort compared to the general population data is due to a phenomenon of research interest—atrophy of the mucosa and its identification depending on the histology of the incisura angularis. It is important to note that patients with autoimmune inflammation are at risk of developing neuroendocrine tumors and, to a lesser extent (compared to atrophic gastritis due to *H. pylori* infection), are at risk of developing gastric adenocarcinoma.

Thus, according to our data, taking into account histological changes in the mucosa in most cases does not have a significant impact on the assessment of the degree and stage of chronic gastritis according to the OLGA system, regardless of the etiology of atrophic gastritis. At the same time, for a cohort of people with a long course of chronic gastritis, with the formation of antralization (severe atrophic changes in the gastric mucosa), taking a biopsy specimen with a subsequent assessment of histological changes in this zone allows the most balanced and accurate assessment of the gastritis grade and stage.

The leading component in assessing the need to take biopsy material from additional points of the gastric mucosa—in particular, from the incisura angularis—is assessing the usefulness/informativeness and cost/workload of this research component. According to the existing data, there is a wide range of reasonable opinions, from ascertaining an insignificant contribution of incisura angularis material to assessing the stage of chronic gastritis to it being a critically necessary component for forming a final conclusion on OLGA classification. Additional informativeness of the use of morphological data when taking material from the incisura angularis may include assessing the topographical features of the gastric mucosa as an additional marker of the evolution of chronic gastritis and the severity of atrophy, the assessment of which requires multicenter studies.

## 5. Conclusions

1. Taking a biopsy from the incisura angularis with subsequent analysis of structural changes in the gastric mucosa in most cases does not play a decisive role in the integral assessment of the stage of gastritis in accordance with the OLGA-system;
2. In persons with a long course of chronic gastritis associated with *H. pylori*, the assessment of the presence and severity of atrophy of the incisura angularis is appropriate for a more accurate assessment of the stage of gastritis.

## Figures and Tables

**Table 1:** Etiology of gastritis in examined patients.

	Etiology	*n*	%
Group 1	Chronic gastritis associated with *H. pylori*	380	52.9
Group 2	Autoimmune gastritis	209	29.1
Group 3	Autoimmune gastritis in combination with chronic gastritis associated with *H. pylori*	129	18.0
	Total	718	100

**Table 2:** Methods for detecting *H. pylori* in groups of patients.

Detection Methods	Group 1. Chronic Gastritis Associated with *H. pylori* (*n* = 380 Patients)		Group 3. Autoimmune Gastritis in Combination with Chronic *H. pylori* Gastritis (*n* = 129 Patients)	
	Total	%	Total	%
^13^C urease breath test	109	28.68%	15	11.62%
Rapid urease test	55	14.47%	26	20.15%
Antibodies to *H. pylori* IgG	64	16.84%	25	19.38%
Morphological study	118	31.05%	4	3.1%
Monoclonal antigen in feces	17	4.47%	3	2.32%
A doctor’s entry in the medical records about the presence of *H. pylori* and the performed eradication therapy	145	38.26%	56	43.41%

**Table 3:** The degree of inflammation of chronic gastritis.

		Grade of OLGA										Total
		0		I		II		III		IV		
		*n*	%	*n*	%	*n*	%	*n*	%	*n*	%	
1	Chronic gastritis associated with *H. pylori*	2	0.5	14	3.7	246	64.7	95	25	23	6.1	380
2	Autoimmune gastritis	1	0.5	2	1	170	81.2	35	16.8	1	0.5	209
3	Autoimmune gastritis in combination with *H. pylori* chronic gastritis	0	-	1	0.8	103	79.8	23	17.8	2	1.6	129
Total		3	0.4	17	2.4	519	72.3	153	21.3	26	3.6	718

**Table 4:** Stages of atrophy of the gastric mucosa in group 1 (chronic gastritis associated with *H. pylori*).

	Stage of OLGA					Total
	0	I	II	III	IV	
Cases considering the incisura angularis	139	98	82	51	10	380
Cases without considering the incisura angularis	155	90	74	55	6	380
Total discrepancies	+16	+8/−15	−8	+4	−4	
Discrepancies with reduced atrophy stage	0	15	8	0	4	27 (7.11%)

**Table 5:** Stages of atrophy of the gastric mucosa in group 2 (autoimmune gastritis).

	Stage of OLGA					Total
	0	I	II	III	IV	
Cases considering the incisura angularis	7	6	35	141	20	209
Cases without considering the incisura angularis	9	6	36	139	19	209
Discrepancies of everything	+2	+2/−2	+3/−2	+1/−3	−1	
Discrepancies with decreasing stage of atrophy	0	2	2	3	1	8 (3.83%)

**Table 6:** Stages of atrophy of the gastric mucosa in group 3 (autoimmune gastritis in combination with *H. pylori* chronic gastritis).

	Stage of OLGA					Total
	0	I	II	III	IV	
Cases considering the incisura angularis	9	7	22	84	7	129
Cases without considering the incisura angularis	10	7	21	85	6	129
Discrepancies of everything	+1	+1/−1	−1	+1	−1	
Discrepancies with decreasing stage of atrophy	0	1	1	0	1	3 (2.32%)

## Data Availability

The data are available upon request to the corresponding author.
